# A novel GMMA-based gonococcal vaccine demonstrates functional immune responses in mice

**DOI:** 10.1038/s41541-025-01190-1

**Published:** 2025-07-05

**Authors:** Marco Spinsanti, Elisabetta Monaci, Giacomo Romagnoli, Giada Buffi, Andrea Guido Oreste Manetti, Filippo Carboni, Giovanna Tuscano, Lucia Eleonora Fontana, Sara Tomei, Marta Zambelli, Rossella Cuffaro, Marianna Taccone, Chiara Sammicheli, Claudia Gianfaldoni, Francesca Angiolini, Maria Giuliani, Sara Marchi, Silvia Senesi, Christian Matano, Ivan Pisoni, Nathalie Norais, Maria Rosaria Romano, Silvia Rossi Paccani, Silvana Savino, Alessandro Muzzi, Federico Fontani, Davide Serruto, Michela Brazzoli, Giulia Giordano, Monica Fabbrini, Ugo D’Oro, Oretta Finco, Immaculada Margarit, Isabel Delany, Erika Bartolini

**Affiliations:** https://ror.org/03fe56089 grid.425088.3 GSK, Siena, Italy

**Keywords:** Vaccines, Bacterial infection

## Abstract

Gonorrhea, caused by *Neisseria gonorrhoeae* (GC) represents a significant public health threat that may be mitigated by an effective vaccine. Vaccines containing *N. meningitidis* outer membrane vesicles (OMVs), such as 4CMenB, demonstrated moderate effectiveness in preventing GC infections. Here, we developed NgG, an investigational GC vaccine based on Generalized Modules for Membrane Antigens (GMMA). NgG includes genetically detoxified OMVs from the FA1090 strain, engineered to reduce endotoxin activity and limit immune interference. NgG induced a robust immune response in mice and outperformed the comparator vaccine 4CMenB in several serological and functional tests. Immunization with GMMA from a FA1090 mutant, where major oligosaccharide epitopes are incomplete or absent, revealed that NgG lipooligosaccharide plays a major role in the breadth of functional responses, with protein component also contributing in some GC strains. These results suggest that NgG has the potential to block GC infection through various mechanisms, supporting further vaccine development.

## Introduction

Gonorrhea, a sexually transmitted infection (STI) that affects both men and women, is caused by the human-restricted pathogen *Neisseria gonorrhoeae* or gonococcus (GC). Typically, gonococcal disease presents as a mucosal infection in the genital tract, rectum, pharynx or eye^1^. It is highly contagious, with many individuals contracting the infection after a single exposure. The World Health Organization (WHO) estimated that over 80 million new cases occurred globally in 2020^2^ and the rate of infections is rising alarmingly. If left undetected or untreated, gonorrhea can lead to serious health complications such as infertility, chronic pelvic pain, ectopic pregnancy, salpingitis, and epididymo-orchitis^1^. Infants born to infected mothers may be underweight or suffer from neonatal conjunctivitis, potentially leading to blindness^3^. Additionally, a GC infection can increase the risk of acquiring and transmitting HIV^4^. The threat posed by GC is exacerbated by its asymptomatic transmission and its ability to repeatedly infect the same individual.

Historically, GC infections have been effectively treated with various antibiotics. However, there is growing concern about the rapid evolution of antimicrobial resistance^5^. The potential for an increasing number of untreatable GC infections led the Center for Disease Control and Prevention (CDC) to rank GC among the top five urgent threats to the US population in 2019^6^. Further, in a global effort to reduce the incidence of sexually transmitted diseases, the WHO has recently set a target goal of 90% reduction of GC incidence by 2030^7^.

Despite 40 years of research on gonococcal vaccines and various candidates under investigation (reviewed in refs. ^8,9^), currently no gonococcal-specific vaccine demonstrating clinical protection is available^10^. Interestingly, vaccines derived from *N. meningitidis* detergent-extracted outer membrane vesicles (OMV) from a New-Zealand outbreak strain, such as MeNZB and 4CMenB (*Bexsero*), have been found in retrospective observational studies to reduce gonorrhea infections with a vaccine effectiveness of 31–40%^11–18^. This could be ascribed to cross-reactive antigens, as GC and *N. meningitidis* present a genome identity ranging between 80 and 90%^19^. OMVs are spherical bi-layered membrane structures derived from the outer membrane of Gram-negative bacteria. They include surface components such as protein and saccharide antigens, as well as self-adjuvanting pathogen-associated molecular patterns (PAMPs), making them particularly attractive for vaccine application^20^. The promising retrospective observational studies on the use of OMV-based meningococcal vaccines has led to renewed hope, for innovative therapeutic or preventive solutions.

With the goal of developing a highly efficacious vaccine in preventing gonorrhea, we investigated a GC-derived OMV vaccine, leveraging on the Generalized Modules for Membrane Antigens (GMMA) technology. GMMA are membrane vesicles from Gram-negative bacteria that are genetically modified to induce hyper-blebbing and/or to show reduced reactogenicity. They can be specifically engineered to deliver a fit-for-purpose vaccine^21,22^. This technology is currently being used in the development of vaccines to combat a range of diseases caused by Gram-negative enteric pathogens, such as *Shigella* and *Salmonella* that have been highlighted as critical by different health organizations^23–25^. Both vaccines are now in the clinical phase, and recently the advanced GMMA-based vaccine to prevent *S. sonnei* infection was shown to be well tolerated and immunogenic in clinical trials in healthy adults and endogenous populations^24,26–28^.

In this manuscript, we present the design and immunogenicity of a GMMA-based GC vaccine (NgG) in preclinical models as well as deconvolution data on the role of main vaccine components (lipooligosaccharide [LOS] sugar and proteins) in functional response. This investigational NgG vaccine is benchmarked in terms of antibody titers and T-cell responses, as well as in functional assays, against the performance of the 4CMenB vaccine in mice.

## Results

### Generation and characterization of the vaccine strain FA1090 Δ*lpxL1*Δ*rmp*

To produce a genetically engineered GMMA vaccine strain from FA1090 gonococcal strain, two genes were targeted for deletion. To reduce the lipid A endotoxin activity, a knockout of the *lpxL1* gene^29^, which is similar to the *Escherichia coli* gene *htrB*, was generated, resulting in the FA1090 Δ*lpxL1* mutant. This gene is responsible for the late acyltransferase phase of lipid A biosynthesis and, when removed, results in penta-acylated instead of hexa-acylated molecules, which reduces the endotoxin activity of the lipid A component of LOS^30–32^. Furthermore, the removal of the reduction modifiable protein antigen (Rmp) was undertaken, as this protein is highly immunogenic and high anti-Rmp antibodies have been reported to correlate with susceptibility of gonorrhea in the clinic and to block complement-dependent killing of GC by otherwise bactericidal antibodies^33,34^. To that purpose, a knockout of the *rmp* gene in the FA1090 Δ*lpxL1* strain was undertaken, generating the GMMA vaccine strain FA1090 Δ*lpxL1*Δ*rmp*.

**Fig. 1:**
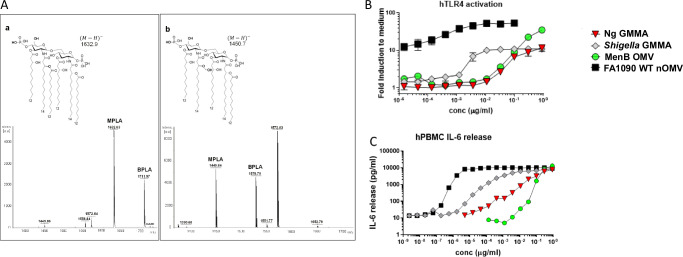
Genetic detoxification assesment. **A** Negative-ion mode MALDI-TOF spectra of the lipid A purified from the WT OMVs (a) and Δ*lpxL1* GMMA (b). The major peaks correspond to monophosphoryl forms of (hexa or penta)acyl-lipid A (MPLA) and diphosphoryl forms of (hexa or penta)acyl-lipid A (BPLA) species. The mass difference between the corresponding peaks in the two spectra is 182.19 Da in agreement with the mass of a lauric acid chain. A signal at m/z 1572.8 corresponding to a non-identified lipid is present in both spectra. **B** Luciferase assay. Serial three-fold dilutions of FA1090Δ*lpxL1*Δ*rmp* (Ng) GMMA, MenB OMV or FA1090 WT nOMV were added to HEK293-hTLR4 cells. The light emitted by luciferase activity was quantified. NF-κB activation of cells stimulated with the different samples was expressed as fold-increase of emitted light over the average result of PBS-stimulated control cells. **C** IL-6 release assay. Purified peripheral blood mononuclear cells (PBMCs) from healthy donors were stimulated with serial three-fold dilutions of Ng GMMA, *Shigella* GMMA, MenB OMV or FA1090 WT nOMV. Released IL-6 was detected in each supernatant by an electrochemiluminescence immunoassay.

The exclusive production of a penta-acylated lipid A molecule after *lpxL1* deletion was verified by mass spectrometry on lipid A extracted from GMMA prepared from the FA1090 Δ*lpxL1* mutant and naturally released OMV (nOMV) obtained from the corresponding wild-type (WT) strain (Fig. 1A). In the MALDI-TOF spectra, lipid A from WT yielded a major molecular ion at *m/z* 1632.03, in agreement with the theoretical mass of hexa-acyl, mono-phosphoryl structure of lipid A (MPLA). In addition to this major form, a diphosphoryl species (BPLA) at *m/z* 1711.97 was also observed. A major component with a molecular ion at *m/z* 1449.84 was detected in the spectrum acquired from the Δ*lpxL1* GMMA lipid A. The difference of mass equal to 182.19 Da observed in comparison with the MPLA form of the WT lipid A is consistent with the lack of a single lauric acid chain (calculated mass: 182.32 Da) in the mutant strain. In addition, the spectrum of the Δ*lpxL1* GMMA also revealed a signal at *m/z* 1529.79 corresponding to the di-phosphoryl form of the lipid A also lacking the lauric acid chain. No signals that could be attributed to the WT form of lipid A were observed.

To further evaluate the efficiency of the GMMA detoxification strategy, two different biological assays were used. First, the biological activity of the mutated LOS on its receptor (TLR4) was evaluated by an in vitro assay using the HEK293-hTLR4 reporter cell line^35,36^. This cell line expresses luciferase under the control of the NF-κB promoter and is stably transfected with human TLR4, MD2, and CD14 complex (HEK293-hTLR4). GMMA prepared from FA1090 Δ*lpxL1*Δ*rmp* (Ng GMMA) were compared with FA1090 nOMV obtained from the wild-type FA1090 WT strain. As a benchmark for the GMMA technology, the *Shigella sonnei* GMMA vaccine^24^ was included in both in vitro assays. As additional comparator, the OMV component (MenB OMV) was also tested. The results of the activation of hTLR4 on HEK293-transfected cells are reported in Fig. 1B, represented as fold induction over the medium-treated cells. Ng GMMA showed lower hTLR4 activation (maximum of 10-fold induction at the highest concentration tested) compared with FA1090 nOMV from the WT strain (maximum of 80-fold induction). No major differences were observed compared with MenB OMV, whereas a lower activity of Ng GMMA was detected compared with *Shigella* GMMA.

The second assay to evaluate the Ng GMMA detoxification strategy consisted of measuring IL-6 release from stimulated human peripheral blood mononuclear cells (hPBMC). IL-6 is a pro-inflammatory cytokine released from hPBMC in large quantity following stimulation with pyrogens and it correlates with pyrogenic effect^37^. The quantification of released IL-6 was carried out with hPBMC from a healthy donor stimulated in vitro with the indicated OMV or GMMA preparations (Fig. 1C). The FA1090 WT nOMV was highly effective at inducing IL-6 release, even when tested at very low concentration (10^−5 ^µg/mL). In contrast, IL-6 release after stimulation with Ng GMMA was dramatically reduced compared with FA1090 WT nOMV and lower compared with *Shigella* GMMA, although slightly higher compared with MenB OMV.

Overall, these data confirmed that the genetic detoxification of the Ng GMMA vaccine component was successful. The comparison with the profiles of two vaccines already tested in humans (*Shigella* GMMA and MenB OMV) supports the prediction of an acceptable in vivo reactogenicity profile in humans.

### Characterization of NgG-induced T-cell responses

**Fig. 2:**
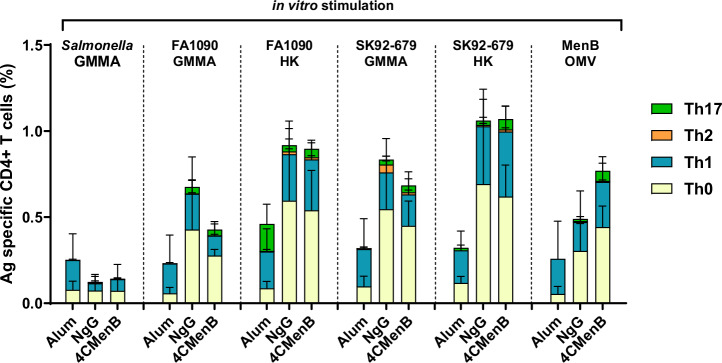
Cellular-mediated immune response analysis. CD1 mice (5 animals/group) were immunized intra-peritoneally, twice, three weeks apart with alum, 4CMenB or NgG. Fourteen days after the second immunization, the frequency of antigen-specific cytokine-secreting CD4^+^ T cells was determined by flow cytometry on splenocytes stimulated in vitro with Salmonella GMMA, FA1090 GMMA, heat-killed (HK) FA1090, SK92-679 GMMA, HK SK92-679, or MenB OMV. Antigen-specific CD4^+^ T cells are expressed as percentage of CD4^+^ T cells. T cell subsets (Th17, Th2, Th1 and Th0) were identified based on the type of secreted cytokines.

To evaluate the T-cell responses induced by NgG (Ng GMMA adsorbed on alum), mice were immunized with alum, NgG, or 4CMenB. Five mice per group were sacrificed on Day 12 after the second immunization and spleens were collected. Splenocytes were stimulated in vitro with the indicated antigens (Fig. 2). Cells producing cytokines were analyzed by flow cytometry and the frequency and quality of the antigen-specific CD4^+^ T cells were determined. In particular, the frequency and the distribution of Th subsets of antigen-specific CD4^+^ T cells upon in vitro stimulation with homologous FA1090 GMMA or the corresponding heat-killed (HK) bacterium (FA1090 HK) were assessed. Moreover, we tested as stimuli the heterologous SK92-679 GMMA (derived from a Δ*lpxL1* mutant), as well as the heat-killed bacterium (SK92-679 HK), and MenB OMV to evaluate the cross-reactivity with a different GC strain or meningococcus stimulation with *Salmonella* GMMA^23^ was used to evaluate the specificity of the response to GC. Data are reported in Fig. 2 and Supplementary Fig. 1. Immunization with NgG induced CD4^+^ T cells cross-reactive against both the homologous and heterologous gonococcal GMMA and bacteria. Notably, CD4^+^ T cells induced by NgG immunization responded to MenB OMV, whereas poor cross-reactivity was observed against *Salmonella* GMMA.

Immunization with 4CMenB induced comparable cellular responses, with CD4^+^ T cells responding to MenB OMV as well as being cross-reactive against gonococcal GMMA and bacteria and poorly recognizing *Salmonella* GMMA.

No major differences were observed in the quality of the response induced by NgG and 4CMenB, with a predominance of Th0 profile, characterized by IL-2 and TNF-α. Low frequencies of IL-17 and IL-4/IL-13-producing cells (Th17 and Th2 profiles) were detected. Background responses were observed in animals treated with alum only, mainly for Th1 CD4^+^ T cells.

### NgG induces systemic and mucosal vaccine-specific antibodies

To evaluate the immunogenicity of NgG and the functional activity of the elicited antibodies, mice were immunized three times on Days 1, 29 and 57 with either of three different batches of NgG, called NgG1, NgG2 and NgG3, or alum alone, or with the 4CMenB vaccine. The three Ng GMMA preparations contained in NgG1, NgG2 and NgG3 exhibited comparable purity levels above 98% (2% of soluble proteins) and a LOS content ranging from 243.8 to 270.1 nmol OS/mg protein, an estimated mean radius of the GMMA being 34.8–35.9 nm and negligible amounts of DNA. Analytical data regarding the GMMA present in the three distinct NgG batches are reported in Supplementary Table 1 and Supplementary Fig. 2.

**Fig. 3:**
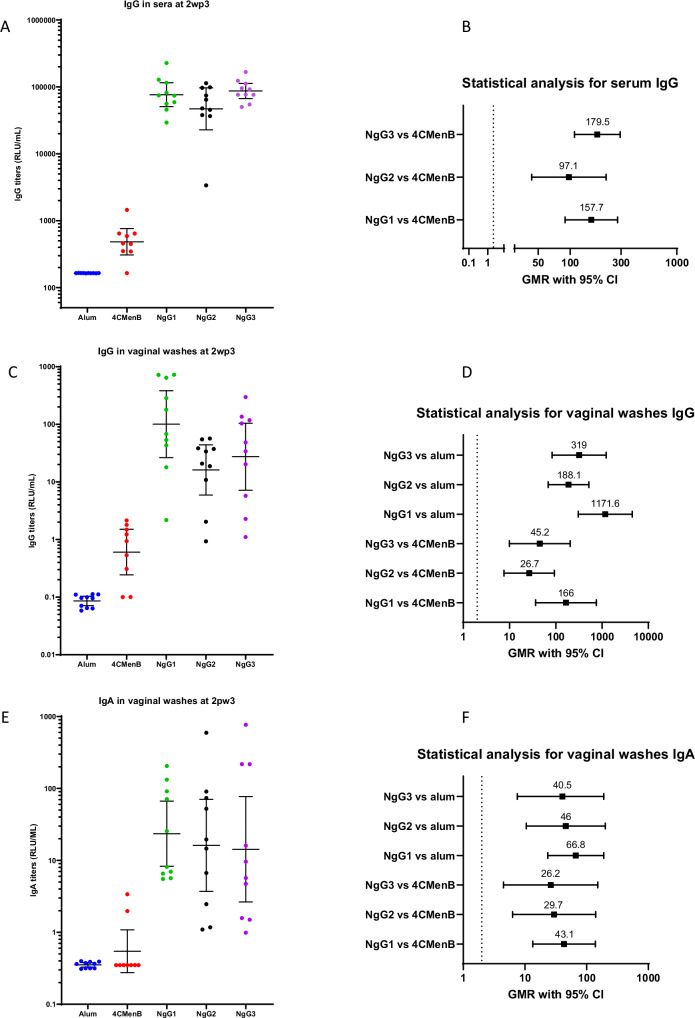
Serum and vaginal antibody response analysis. CD1 mice (10 animals/group) were immunized three times intra-peritoneally on Days 1, 29 and 57 with alum, 4CMenB or NgG (three different batches, NgG1, NgG2 and NgG3). Two weeks after the third immunization (2wp3), sera and vaginal washes were collected and the specific anti-Ng GMMA antibody responses were measured by Luminex assay. **A** Individual IgG serum titers for each immunization group are shown, with mean and 95% confidence intervals. **B** The statistical analysis for individual IgG serum titers is shown as geometric mean ratio (GMR) comparison (with 95% confidence intervals) for each batch versus 4CMenB. **C** Individual IgG vaginal titers for each immunization group are shown, with mean and 95% confidence intervals. **D** The statistical analysis for individual IgG vaginal titers is shown as geometric mean ratio (GMR) comparison (with 95% confidence intervals) for each batch versus alum and 4CMenB. **E** Individual IgA vaginal titers for each immunization group are shown, with mean and 95% confidence intervals. **F** The statistical analysis for individual IgA vaginal titers is shown as geometric mean ratio (GMR) comparison (with 95% confidence intervals) for each batch versus alum and 4CMenB. For all statistical results, the dotted lines at x = 2 indicate the threshold for the Lower Limit of GMR beyond which the differences are significant.

The analysis of the antibody responses was performed in sera and vaginal washes collected two weeks post-third immunization (2wp3) and measured by Luminex assay. For all three NgG batches, single 2wp3 sera showed about two log-fold higher anti-Ng GMMA serum IgG titers, compared with those obtained when immunizing with 4CMenB vaccine or alum alone (Fig. 3A). A statistical analysis taking into consideration GMR with 95% confidence interval (CI) confirmed the superiority of all three NgG batches compared with 4CMenB (Fig. 3B). Anti-Ng GMMA IgG titers in individual vaginal washes from immunized mice were higher than those induced by alum or 4CMenB vaccine, showing a lower limit (LL) of GMR ≥ 2 (Fig. 3C, D). As IgA plays a relevant role in mucosal immunity, specific anti-Ng GMMA IgA were also measured in vaginal washes. The three groups immunized with NgG showed the highest IgA titers, with LL of GMRs ≥2, compared with both alum and 4CMenB (Fig. 3E, F).

### NgG induces antibodies capable of complement-mediated bacterial killing

**Fig. 4:**
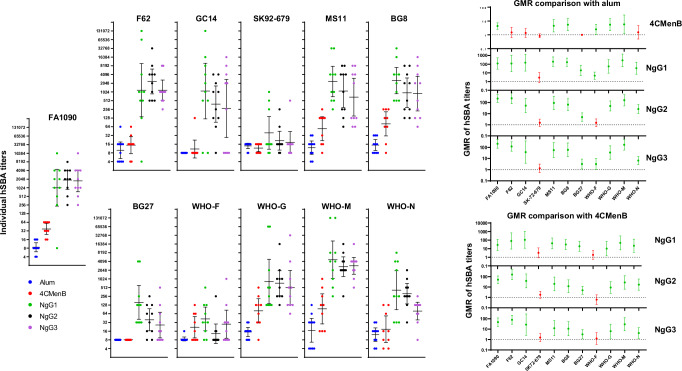
Human serum bactericidal assay (hSBA) results. Mice were immunized with alum, 4CMenB or NgG (three different batches called NgG1, NgG2, or NgG3). Sera were collected two weeks after the third immunization and functional antibodies were measured by hSBA against the homologous FA1090 GC strain and the heterologous BG27, BG8, F62, GC14, MS11, SK-92-679, WHO-F, WHO-G, WHO-M and WHO-N strains, using normal human serum from healthy donors as complement source. On the left side of the panel, individual serum titers are shown for each GC strain. On the right are shown statistical results as geometric mean titers (GMR) comparison (with 95% confidence intervals) between each strain and alum (upper part) and each strain and 4CMenB (lower part). The dotted lines at y = 1 indicate the threshold for the Lower Limit of GMR beyond which the differences are significant. Non statistically superior results are shown in red.

A human serum bactericidal assay (hSBA) was used to assess the presence of immunoglobulins in the serum of NgG-immunized mice, able to kill bacteria by activating the human complement cascade. In addition to hSBA against the homologous FA1090 strain, the analysis was extended to a panel of 10 heterologous strains in order to assess potential vaccine cross-functional activity. These heterologous strains were selected for being representative of the heterogeneity of the GC population, based on the genetic variability of the 59 more abundant proteins identified by mass spectrometry analysis of Ng GMMA (Supplementary Table 2). The hSBA analysis was performed on single sera from animals receiving three doses of either NgG1, NgG2, NgG3, 4CMenB or alum. To develop specific hSBA assays, we evaluated each of the 11 Ng strains tested in this study for their sensitivity to complement-mediated lysis using non-immune human serum as the complement source. Four out of eleven strains were identified as serum-sensitive and cultivated in a medium containing Cytidine-5’-*O*-monophospho-*N*-acetylneuraminic acid (CMP-NANA) at the minimum concentration necessary to confer serum resistance. Four out of eleven strains were classified as serum-sensitive and therefore were tested in hSBA after growth in medium containing CMP-NANA. Results are presented in Fig. 4. A statistical analysis taking into consideration the LL of 95% CI of GMR showed significant differences when the 4CMenB group was compared with alum for 6/11 tested strains. Notably, sera from mice receiving any of the three NgG batches showed statistical superiority (LL 95%CI of GMRs ≥1) compared with both alum and 4CMenB against all tested strains except SK92-679 and WHO-F, for which only few sera had bactericidal activity (Fig. 4). SK92-679 and WHO-F belong to Cluster 1 which is particularly heterogeneous, and they do not belong to a specific subcluster. Overall, the three NgG batches outperformed 4CMenB with GMRs ranging from 3.2 for NgG3 on BG27 strain to 158.8 for NgG2 on F62 strain.

### NgG induces antibodies capable of inhibiting adhesion to two human epithelial cell lines

The ability of NgG to elicit anti-NgG serum antibodies capable of inhibiting bacterial adhesion to epithelial cells was assessed using a cell-based fluorescent bacterial adhesion inhibition assay on the homologous FA1090 strain and on the heterologous SK92-679 strain with two human epithelial cell lines representing male and female urogenital epithelial cells (SV-HUC1 ureteral cells, Ect1 ectocervical cells). As negative control, sera obtained from mice immunized with alum were used, whereas sera raised against 4CMenB were used as comparator.

**Fig. 5:**
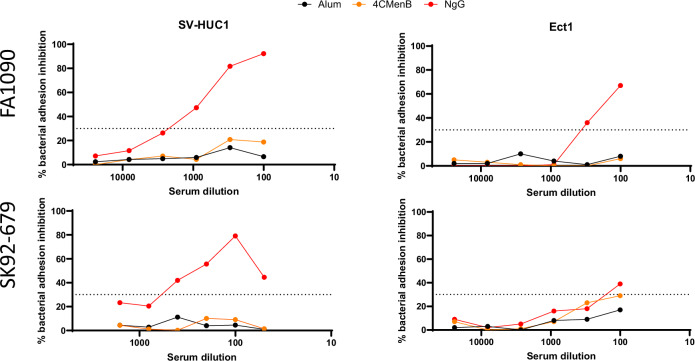
Bacterial adhesion inhibition assay (BAI). In this assay, the capacity of alum-, 4CMenB- and NgG-immunized murine sera to inhibit the adhesion of homologous FA1090 and heterologous SK92-679 GC strains to epithelial cells was assessed. Two different epithelial cell types were used: SV-HUC1 human ureteral cells and Ect1 human ectocervical. Briefly, GC strains (FA1090 or SK92-679) were labeled with Oregon Green dye before incubation with serially diluted murine sera (pooled 2wp3 sera). Bacteria-sera complexes were then added to the cultured epithelial cells. The percentage of adhesion inhibition was evaluated after one hour. The dotted lines at 30% indicate the blank limit.

Pooled 2wp3 sera collected from NgG-immunized mice showed a titratable capacity of inhibiting adhesion of the homologous FA1090 strain to both epithelial cell lines (Fig. 5), reaching up to 67–92% inhibition at the lowest serum dilutions. Adhesion of the heterologous SK92-679 strain to the SV-HUC1 and Ect1 cells was also inhibited by NgG-immunized sera (Fig. 5). In contrast, no inhibition was observed with anti-4CMenB sera (<30%, blank limit), be it with FA1090 or SK92-679 (Fig. 5) even if a borderline result was observed for the SK92-679 with Ect-1 cells.

### Anti-LOS antibodies play a variable role in the functional responses to NgG depending on the strain

NgG contains multiple immunogenic components including many outer membrane proteins and LOS. *N. gonorrhoeae* produces LOS which makes up to roughly 50% of the mass of the outer membrane of the bacterium^38^. LOS is an important gonococcal virulence factor and plays a key role in several aspects of gonococcal pathogenesis, including but not limited to resistance to complement, adhesion and entry into cells and recognition by the innate immune system^39^. Antibodies directed against LOS activate the complement system, resulting in direct killing of *N. gonorrhoeae*^40^. This has prompted efforts to evaluate LOS as a vaccine candidate. Antibodies against LOS may protect against re-infection with the homologous strain, as suggested in a male urethral experimental gonococcal infection model^41^. To assess the role of the Ng GMMA surface-exposed OS and of the protein components in the functional immune response against NgG, we generated in the background of the vaccine strain FA1090 Δ*lpxl1*Δ*rmp* the FA1090 Δ*lpxl1*Δ*rmp*Δ*lgtF* mutant strain lacking *lgtF*, a key LOS biosynthetic gene. The *lgtF* gene encodes for a glucosyltransferase responsible for the extension of the α-chain from the core heptose HepI. Therefore, complete removal of α-chain leads to a mutant strain in which all OS epitopes targeted by the majority of anti-LOS antibodies are incomplete or absent^42–44^. LOS migration of the GMMA extracts from the obtained mutant in a silver-stained SDS-PAGE and parallel MS analysis confirmed the α-chain loss, and suggested that it predominantly lacked the Hep2 substitution (data not shown). Indeed, we confirmed that the LOS from the FA1090 Δ*lpxl1*Δ*rmp*Δ*lgtF* mutant strain was not recognized by the 2C7 monoclonal antibody (Supplementary Table 1). A Coomassie blue-stained SDS-PAGE gel (Supplementary Fig. 2) indicated that FA1090 Δ*lpxl1*Δ*rmp*Δ*lgtF* GMMA (Ng GMMA Δ*lgtF*) presented similar protein profile as for Ng GMMA, except for few bands that were ascribed to cytoplasmic contamination; further, a more precise relative quantification of the surface proteins present in the two GMMA performed by mass spectrometry revealed that the 28 most abundant proteins in GMMA from Δ*lgtF* mutant (representing up to 97% or the total amount) were also present in similar abundance in the Ng GMMA.

The 2C7 epitope is a conserved oligosaccharide structure, part of LOS on GC, composed by two lactose units, respectively linked to Hep I and Hep II. The LOS epitope recognized by the mAb 2C7 has been described as essential for anti-LOS activity^45^. Furthermore, the 2C7 monoclonal antibody reacts with 95% of fresh clinical isolates, and it enhances clearance of GC infection in a mouse lower genital tract infection model^46^, suggesting that such phase variable antigens might be effective vaccine targets. The 2C7 epitope has been examined as a potential gonococcal candidate.

**Fig. 6:**
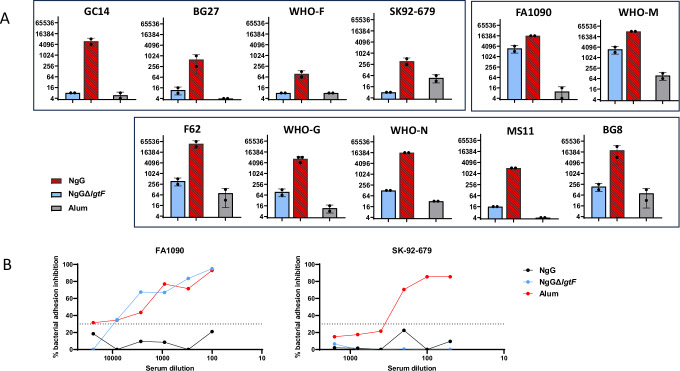
Role of anti-LOS antibodies in the functional response to the NgG vaccine. Mice were immunized with alum, NgG or NgGΔ*lgtF*. **A** Sera were collected two weeks after the third immunization and functional antibodies were measured by hSBA against the homologous FA1090 strain and the heterologous BG27, BG8, F62, GC14, MS11, SK92-679, WHO-F, WHO-G, WHO-M and WHO-N strains, using normal human serum from healthy donors as complement source. Titers obtained with pooled sera are shown for each GC strain. **B** The capacity of alum-, NgG- and NgGΔ*lgtF*-immunized murine sera to inhibit the adhesion of homologous FA1090 and heterologous SK92-679 strains to SV-HUC-1 epithelial cells was assessed. Briefly, GC strains (FA1090 or SK92-679) were labeled with Oregon Green dye before incubation with serially diluted murine sera (pooled 2wp3 sera). Bacteria-sera complexes were then added to the cultured epithelial cells. The percentage of adhesion inhibition was evaluated after one hour. The dotted lines at 30% indicate the blank limit.

The hSBA titers induced in mice by NgG or NgGΔ*lgtF* (Ng GMMA Δ*lgtF* adsorbed on alum) were measured in pooled sera against FA1090 and the 10 heterologous strain panel. From Fig. 6A, which compares the hSBA of NgG and NgGΔ*lgtF*, it is clear that the involvement of LOS-specific antibodies elicited by NgG in bactericidal activity was variable, depending on the target strain. Indeed, anti-NgG sera mediated bactericidal killing of all 11 evaluated strains (ratio SBA titers vs alum negative control >4, positive threshold established based on assay variability). Importantly, in the former analysis shown in Fig. 4, only few anti-NgG individual sera showed bactericidal activity against SK92-679 and WHO-F. In contrast, here, anti-NgG pooled sera showed positive bactericidal activity against the two strains, even though it was with low titers (8- and 12-fold above alum respectively). Conversely, 7/11 strains killed by anti-NgG sera were not killed by anti-NgGΔ*lgtF* sera, indicating an exclusive functional role of the anti-LOS antibodies. Among the remaining four strains, two (FA1090 and WHO-M) showed high susceptibility to killing by anti-NgGΔ*lgtF* sera with bactericidal titers comparable to anti-NgG, indicating a major role of antibodies induced by the protein component of GMMA, while for the other two (F62 and WHO-G) killing of NgG antisera was more than 64-fold higher than the killing induced by NgGΔ*lgtF* antisera, suggesting both anti-protein and anti-LOS antibodies contribute to bactericidal activity.

Since it is well known that glycans can induce sizeable IgM responses that mediate strong bactericidal activity^47^, we sought to test whether IgM-depleted immune sera of NgG-immunized mice still presented functional activity. Pooled sera were depleted of IgM as described by Gulati et al.^48^ and were tested in hSBA against two gonococcal strains (F62 and WHO-N) selected for the important contribution of anti-LOS antibodies to their killing. For both strains, we observed that SBA titers were reduced by almost ten-fold after IgM depletion (from 10^4^ to 10^3^), but remained ten-fold higher than those of the negative control (10^2^), suggesting that both IgM and IgG had an important effect on the functional activity elicited by the NgG vaccine.

The potential role of anti-LOS and anti-protein antibodies was also assessed with the BAI assay (Fig. 6B) on two strains. The sera raised against either NgGΔ*lgtF* or NgG were similarly able to inhibit the binding of the homologous FA1090 strain to SV-HUC1 epithelial cells in a dose-dependent manner (*p* ≤ 0.05), whereas alum-immunized sera did not. This result is indicative of a major contribution of anti-protein antibodies for the recognition and inhibition of the FA1090 homologous strain. In contrast, the LOS component appeared as a major antibody target for the inhibition of SK92-679 strain adhesion to that same epithelial cell line (Fig. 6B), as the antisera generated by NgGΔ*lgtF* immunization did not exhibit any inhibition on this strain.

In conclusion, the functional activity of antibodies elicited by both protein and LOS components of NgG have distinct and broad activity across a diverse strain panel through multiple mechanisms, including complement-mediated killing and bacterial adhesion inhibition to mucosal models.

## Discussion

A highly effective vaccine against gonococcal infections is a pressing need^49^. After the promising findings from OMV-based meningococcal vaccine effectiveness on gonorrhea, there has been renewed interest in a GC vaccine. Here, research efforts were directed towards the development of a gonococcal vesicle vaccine based on GMMA technology. To achieve this, a double mutant strain was successfully engineered in the GC FA1090 genetic background, removing the *lpxL1* and *rmp* genes. The GMMA from this mutant strain harbored a genetically detoxified LOS with fully penta-acylated lipid A, and our data indicates that the *lpxL1* genetic detoxification effectively reduced hTLR-dependent activation and IL-6 release from human PBMC by GC. This was an important step in the pursuit of a GMMA-based gonococcal vaccine. A similar genetically engineered detoxification strategy has given rise to well-tolerated penta-acylated meningococcal OMV vaccines^50^, as well as *Shigella* GMMA vaccine^27^, both with acceptable reactogenicity profiles in humans.

A second mutation aimed to remove the Rmp antigen, for which the immunity in humans has been correlated with an increased susceptibility to gonorrhea through a possible mechanism of blocking antibodies^33,34,51,52^. Studies in mice have further confirmed that immunization with OMV derived from an Rmp-mutant GC results in higher bactericidal antibody responses, and active immunization with Rmp or passive transfer of anti-Rmp antibodies to mice prolongs infection after challenge with GC^33,51^.Therefore, a rationale for creating Rmp-free GMMA was to achieve a higher vaccine efficacy. Interestingly, the deletion of *rmp* in the vaccine strain resulted in an increased GMMA yield (data not shown), likely through partial destabilization of the outer membrane resulting in overblebbing, as has been reported in meningococcus^53^.

Previously, gonococcal OMV-based investigational vaccines, when administered with IL-12 by intravaginal or intranasal routes, have demonstrated effectiveness against challenge in mice with diverse strains of GC, including an OMV from a Δ*lpxL1*Δ*rmp* double knock-out strain^54,55^. Interestingly, parenteral immunization with the 4CMenB vaccine led to protection in similar estradiol-treated mouse challenge models^56^. Here, we assessed the adaptive immune response of NgG after parenteral immunization in mice and evaluated the functional activity of the antibodies generated by NgG immunization against a panel of GC strains, comparing with the responses elicited by the 4CMenB comparator vaccine.

Human studies have shown that natural infection often induces a limited immune response, with low antibody responses^57^, which may at least partly explain why this pathogen is able to re-infect the same individual with little evidence of protective immunity from infection^58^. The lack of protection may be due to the capacity of the bacteria to escape the immune system as well as the failure of the host to mount an efficient protective immune response. GC is indeed known to possess several mechanisms to impede the host defense responses, both indirectly through antigenic variation mechanisms and directly through dampening of adaptive and innate immune responses^10,58^. For example, recent evidence suggested that GC selectively induces a Th17-driven response during infection and is able to suppress the protective cell-mediated Th1 and Th2 immune responses^10,58,59^. The fact that OMV-based MenB vaccines (including 4CMenB) were recently shown to cross-protect against GC^11–18^ highlighted the possibility to induce immune protection against gonococcus and a potential mechanism underlying cross-protection may include the induction of T-cell responses and antibodies against shared antigens^60^.

Although GC is a human-restricted pathogen, studies using mouse models provide useful insights into the immune response to gonorrhea. Studies in mice highlighted that Th1-mediated immunity was associated with accelerated gonococcal clearance and resistance to reinfection^59,61^. Moreover, it was shown that blocking regulatory cellular responses with anti-IL-10 or anti-TGF-β monoclonal antibody resulted in increased protection in a mouse model of infection^61,62^. These data support the evidence that a contribution of cellular-mediated immunity is important not only to sustain the humoral immune response but also to provide the best cytokine environment to control gonococcal infection. We demonstrated that NgG efficiently promotes the induction of antigen-specific CD4^+^ T cells in CD1 mice, with a Th0 phenotype. Induced CD4^+^ T cells were broadly cross-reactive, recognizing a homologous and a heterologous strain, as well as MenB OMV, suggesting the presence of common T-cell epitopes among *Neisseria* species. Interestingly, no cross-reactivity was observed against *Salmonella* GMMA.

Taking into account the different pathogenic mechanisms for GC infection, it is expected that a successful vaccine against GC would generate antibodies able to induce complement-mediated killing and/or to block bacterial adhesion. Therefore, a human serum bactericidal assay and a bacterial adhesion inhibition assay to human mucosal cells were developed to evaluate the functional activity of the antibodies generated by NgG immunization against GC strains. When injected in mice, NgG elicited high levels of GC GMMA-specific serum IgG, exceeding by about two-log10 factors the levels obtained after immunization with 4CMenB. The IgA responses measured in NgG-immunized mice suggested that mucosal immunity was induced via parental immunization. IgG and IgA titers in vaginal washes, where GC more specifically needs to be targeted, were also much higher after immunization with NgG than after 4CMenB. Not only was NgG able to induce a robust antibody response, but these antibodies were also shown to be bactericidal towards a large panel of GC strains, encompassing genetically close and remote strain clusters versus the homologous FA1090 vaccine strain. Bactericidal antibodies have long been established as a correlate of protection against meningococcal infections^63^, but a correlate of protection against gonococcal infection has yet to be defined. Nevertheless, there is evidence suggesting that complement-mediated killing may be an important mechanism of protection from gonococcal disease in recent mice studies^64,65^. Among the tested strains, two were less efficiently killed in the bactericidal assay, namely WHO-F and SK92-679. However, the anti-NgG antibodies were able to efficiently inhibit the adhesion of SK92-679 to both male and female urogenital epithelial cells in the BAI assay, suggesting that NgG elicits distinct subsets of antibodies with specific functionality. Our functional assays were performed on GC mainly without sialylation, whereas LOS sialylation occurs in vivo, which may represent a limitation of our study. However, the levels of gonococcal sialylation can vary significantly depending on several factors, including strain variations, environmental conditions and local sialylation dynamics between male and female niches^66^.

Several mucosal tissues, but more particularly the epithelium of the lower and upper genitourinary tracts of both men and women is the primary site of colonization by GC^67^. Mucosal antibodies able to inhibit the adhesion of GC to the epithelium of the urogenital niche may be instrumental for blocking colonization and invasion. In previous human studies, it has been reported that pilus-specific antibodies that blocked gonococcal adherence to human mucosal cells appeared to correlate with protection^10,68^. This was evidenced by the high inhibitory activity measured in vaccinees against the homologous vaccine strain, and protection was afforded upon challenge with this strain even if the vaccine did not protect against heterologous strains or in the field^68^. By using a BAI assay the capability for the NgG vaccine of inducing antibodies to prevent bacterial adhesion to two human cell lines representative of the male and female urogenital epithelium was demonstrated and the adhesion of the homologous FA1090 was effectively blocked to both mucosae in the presence of anti-NgG antibodies. Anti-4CMenB antibodies did not show inhibition against FA1090 in this assay. Due to the multiantigen nature of NgG, antibodies raised against different antigens may be responsible for inhibiting adhesion. While there is no measurable pilus protein in NgG (Supplementary Table 2), LOS, Opa proteins or other adhesins may be implicated in eliciting antibodies able to block host-pathogen interactions in the mucosae. It is noteworthy that the anti-NgG antisera successfully inhibited SK92-679 bacterial adhesion but was not bactericidal in hSBA toward the SK92-679 strain. Anti-LOS antibodies were implicated in the inhibition of adhesion of the SK92-679 strain to male cell mucosae, as no inhibition was observed with antisera elicited by NgGΔ*lgtF* lacking all major OS epitopes. The host-pathogen interactions important for GC infection of the urogenital mucosae are known to differ between men and women^69^, with LOS being an important ligand for asialoglycoprotein receptor in the male^70^ and Opa proteins being important for CEACAM interactions in the female mucosal niche^71^. Our data suggest, perhaps counterintuitively, that different subsets of NgG-induced antibodies may have different functionality against different strains, as highlighted by the positive BAI but negative hSBA activities towards the SK92-679 strain.

LOS is the most abundant gonococcal outer membrane molecule, playing a major role in gonococcus pathogenesis and has for long been identified as a major gonococcal antigen^72,73^. Given the potential importance of LOS in the generation of protective immunity against gonococcus^74,75^, we further investigated the role of anti-LOS functional immune responses induced by NgG. Immunization of mice with a GMMA void of the majority of LOS oligosaccharide epitopes (NgGΔ*lgtF*) generated sera with negative hSBA titers against six heterologous strains or decreased titers against three other heterologous strains compared with immunization with NgG. This indicates the involvement of gonococcal LOS in inducing cross-bactericidal functional responses. On the contrary, high bactericidal responses were measured for the FA1090 homologous strain and WHO-M heterologous strain from the NgGΔ*lgtF-*generated antisera, implying that the protein component would be responsible for the majority of bactericidal responses against these specific strains. To summarize, our data suggest a fundamental role of gonococcal LOS at inducing bactericidal and adhesion-inhibiting antibodies able to target heterologous strains, although a major contribution of the proteins expressed on GMMA surface for the cross-functional responses is evident for certain strains, including the homologous strain. Overall, these observations reflect the great versatility of the multiantigen NgG.

In conclusion, the investigational NgG demonstrated potential as a gonococcal vaccine. Parenteral immunization of mice resulted in a strong Th0-skewed CD4^+^ T-cell response quite similar to that of the 4CMenB comparator. However, NgG outperformed 4CMenB in the induction of GC-binding serum and mucosal IgG and IgA. It was also superior to 4CMenB at inducing functional responses, as shown in two distinct important assays with human complement or human cells, which may be important for protective immunity. Based on these promising results, the development of this vaccine has been pursued and is under investigation in the clinical development phase (ref Clinical trial.gov NCT05630859).

## Materials & methods

### Generation of recombinant gonococcal strains and GMMA thereof

The parent isolate for preparation of the vaccine strain was FA1090 derived from a patient with disseminated invasive gonorrhea^76^. The *lpxL1*, *rmp* and *lgtF* genes of FA1090 were respectively deleted through homologous recombination with *pBS-*Δ*lpxL1 kanR*^77^, pBS-Δ*rmp eryR* and pBS-*lgtF::cmR* constructs containing upstream and downstream regions of the genes flanking an antibiotic resistance cassette. All polymerase chain reactions (PCR) were performed with the KAPA Hifi 2X master mix (Roche) and primers reported in Supplementary Table 3. For the generation of the pBS-Δ*rmp eryR* construct, amplification of the up and downstream regions of the *rmp* gene was performed with the primer couples *UpIII-FOR/REV* and *DpIII-FOR/REV* using 50 ng of genomic DNA of the FA1090 strain as template. Amplification of the erythromycin resistance gene (*eryR*) was done with the primer couple *EryR_gono_SmaI-Fw*/*Rev* using as template 10 ng of a plasmid carrying the gene. PCR products were purified using the kit Wizard SV Gel and PCR Clean-Up System (Promega), according to the manufacturer’s instructions, and cloned as *XbaI-SmaI* (UP *rmp*), *SmaI* (eryR) and *SmaI-XhoI* (DOWN *rmp*) into a pBluescript vector digested with *XbaI-XhoI* (NEB). Amplification of upstream and downstream regions of the *lgtF* gene was performed with the primer couples *lgtF-UP-fw*/*rv* and *lgtF-DO-fw*/*rv*, respectively, using 50 ng of genomic DNA as template; the amplification of the chloramphenicol resistance gene (*cmR*) was done with the primer couple *cloKOF*/*R* using 10 ng of a synthetic DNA template (Geneart). PCR products were transformed into *E. coli* MACH-1 competent cells (Thermo Scientific) immediately following amplification. Screening of transformants was done by colony PCR using *T7prom*/*pETseqRv* primers. The PCR product amplified using the primers *Lpx UP Fwd* and *LpxL1 DO Rev* and the plasmid *pBS-*Δ*lpxL1 kanR* as a template, the pBS-Δ*rmp eryR* linearized plasmid or the PCR product of the positive *lgtF* clone were used for the transformation of the FA1090, the FA1090 Δ*lpxL1* or the FA1090 Δ*lpxL1*Δ*rmp* strain, respectively. Transformations were carried out by spotting a mixture of 30 µl of bacterial resuspension in phosphate-buffered saline (PBS) and 30 µl of DNA onto a GC agar +1% isovitalex plate for 5–6 h and transformants were selected on GC agar +1% isovitalex plates with either kanamycin 40 µg/ml, erythromycin 2 µg/ml or chloramphenicol 2 μg/ml. All transformants were tested by PCR analysis using Accuprime Taq Polymerase (Thermo Scientific) and with external primers (primer couples *LpxL1 est FW*/*REV*, *UP_CHECK_NGO1577-Fw*/*DW_CHECK_NGO1577-Rev*, and *lgtF-ext-F*/*R* for Δ*lpxL1*, Δ*rmp* and Δ*lgtF*, respectively) to check the correct event of double recombination. The genome of the final FA1090 Δ*lpxL1*Δ*rmp* vaccine producer strain was obtained using both Illumina and PacBio sequencing and checked with respect to regions relative to the gene deletions.

To generate small scale GMMA or nOMV preparations, cell-free supernatants were recovered by centrifugation at 8000 × *g* for 15 min at 4 °C from overnight cultures of the gonococcal WT or mutant strains in Meningitis Chemically-defined Medium I (MCDMI-mod, composed as follows: 15 g/L Soy peptone (BBL Phytone), 5.8 g/L NaCl, 2.56 g/L MgSO_4_–7H_2_0, 5.24 g/L K_2_HPO_4_–3H_2_0, 3 g/L L-Glutamic Acid, 0.20 g/L L-Arginine, 0.5 g/L L-Serine, 0.3 g/L L-Cysteine, 0.25 g/L L-Glycine, 0.01 g/L Fe(III) Citrate, 0.02 g/L CaCl_2_, 5 g/L Na-(DL)Lactate, 0.34 g/L Betaine-H20, 0.5 g/L Vitamin Mix(5X) consisting of 0.5 g/L Thiamine, 0.5 g/L Riboflavin, 0.5 g/L Pyridoxine, 0.5 g/L Niacinamide) at 37 °C and filtered through 0.2-μm Sartobran P H9 filter to ensure bacteria removal. After filtration, MgCl_2_.6H_2_O was added to a final molarity of 1 mM and 50 U/L of benzonase was added for DNA removal and incubated at 4–8 °C with stirring overnight. A tangential flow filtration step using 300 K Sartocon slice cassettes was used for retention of the GMMA and buffer exchange in PBS, followed by an ultracentrifugation step (30,000 × *g* for 15 h) to pellet the GMMA which were subsequently resuspended in PBS and sterile-filtered using a 0.2 µm filter, and the product was stored at –20 °C.

To generate the three batches of Ng GMMA from FA1090 Δ*lpxL1*Δ*rmp* strain Ng GMMA 1, Ng GMMA 2 and Ng GMMA 3, medium scale preparations were performed. One Erlenmeyer flask containing MCDMI-mod, pH 6.9 was inoculated with a vial of strain FA1090 Δ*lpxL1*Δ*rmp* seed preparation and incubated at 37 °C, under agitation. When the culture reached exponential growth, a fermenter containing 1.5 L of MCDMI-mod medium was inoculated with a starting optical density at 590 nm (OD_590_) equal to 0.3–0.4. The fermentation occurred under the following conditions: temperature 37 °C, air flow rate 1 vvm, pH 6.9 controlled through concentrate acid as titrant, dissolved oxygen tension (DOT) set point 30% controlled through impeller stirring speed. Upon depletion of carbon sources in the medium, the supernatant was recovered by centrifugation (8000 × *g* for 15 min at 4 °C) and filtered through 0.2 μm, to ensure bacterium removal. One mM MgCl_2_ and 50 kU/L benzonase were added to hydrolyze DNA potentially released in the supernatant. The material was then purified through tangential flow filtration (300 KDa PES cassettes with around 50 kg supernatant/m^2^ of membrane surface) and the diaretentate was eluted from a chromatography step, using a multimodal resin (CaptoCore 700) in buffer PBS pH 7.4. Total protein content was estimated by the Lowry method, and the product was stored at –70 °C after a final 0.2 μm filtration.

### Characterization of GMMA

Total protein content of GMMA was estimated by the Lowry method. The purity of Ng GMMA and Ng GMMA Δ*ltgF* were evaluated by sodium-dodecyl-sulfate polyacrylamide gel electrophoresis (SDS-PAGE) and size exclusion chromatography module (SE-HPLC). In particular, SE-HPLC checked the purity of GMMA preparations, allowing determination of generally occurring contaminants such as soluble proteins (reported as fluorescence (FLR)) and DNA (reported as µg DNA/µg protein).

Multiangle light scattering (MALS) coupled with high-performance liquid chromatography-size exclusion chromatography (SEC) was used to assess GMMA particle radius. The LOS content in Ng GMMA was measured by SE-HPLC analysis by quantifying the reactive carbonyl groups of the saccharide moiety (OS) generated after acid hydrolysis to remove the lipid A and derivatized with semicarbazide, as previously reported^78^. LOS was characterized by immunoblotting using murine mAb 2C7 as the antibody^79^. Lipid A was extracted from Ng GMMA using mild-acid hydrolysis and analyzed with an Ultraflex matrix-assisted laser desorption ionization–time of flight (MALDI-TOF) mass spectrometer (Bruker Daltonics) in negative-ion reflectron mode as previously reported^78^.

### Luciferase assay with hTLR4-transfected HEK293 NF-κB-reporter cell lines

HEK293-hTLR4-transfected adherent cells were maintained in Dulbecco’s modified Eagle medium (DMEM) supplemented with 4.5 g/l glucose and HEPES (GIBCO), 10% fetal bovine serum (FBS, Hyclone), 1% penicillin/streptomycin/glutamine, and specific antibiotics for the different cell lines: puromycin (5 µg/ml Invivogen), blasticidin (10 µg/ml; Invivogen), hygromycin (250 µg/ml, Roche). For the NF-κB luciferase assay, 25,000 cells/well were seeded in 90 µl of complete DMEM without antibiotics in 96-well μClear luciferase plates (Greiner Bio) and incubated for 24 h at 37 °C, 5% CO_2_ before 10 µl of serial 3-fold dilutions of the different samples were added. After incubation for 5 h at 37 °C, supernatants were removed by aspiration, and cells were lysed for 20 min at room temperature (RT) using 20 µl/well of 1:5 diluted Passive Lysis Buffer (Promega). Eleven three-fold dilutions of tested samples were prepared in PBS from a serial dilution starting at 9 µg/ml for Ng GMMA, *Shigella sonnei* GMMA (kindly provided by the GSK Vaccines Institute for Global Health, Siena, Italy) and MenB OMV (detergent-extracted OMV from meningococcus NZ98/254 strain from commercial 4CMenB vaccine – GSK batch product), 1 µg/ml for FA1090 WT nOMV (native OMV). Ten microliters of serially diluted stimuli were added to the cells in a final volume of 100 µl (final concentration 0.9 and 0.1 µg/ml). A negative control (PBS only) and a positive control (USP Reference Standard Endotoxin, RSE) were included in each plate. Luciferase activity was detected using 100 µl/well of Luciferase Assay Reagent (Promega), and emitted light was immediately quantified using a Tecan Spark luminometer.

### IL-6 release assay

Purified PBMC from healthy donors were plated in a 96-well plate at the concentration of 1.5 × 10^5^ cells/well in RPMI supplemented with 100 U/ml penicillin, 100 µg/ml streptomycin, 10 mM HEPES, minimum essential medium with non-essential amino acids, 2 mM L-glutamine and 1% (v/v) FBS. Three-fold dilutions of the same OMV and GMMA samples as in the previous assay were prepared in PBS from a starting concentration equal to 9 μg/ml. Amount of 20 μl of the stimuli were added to the cells in a final volume of 200 μl (final concentration 0.9 μg/ml). PBMC of the selected donors were incubated overnight with the stimuli at 37 °C in a humidified atmosphere containing 5% CO_2_. Quantitative determination of IL-6 in PBMC supernatants stimulated with different samples was performed using an electrochemiluminescence immunoassay system from Meso Scale Discovery (MSD) platform according to the manufacturer’s instructions.

### Immunogenicity in animal models

Animal husbandry and experiments were ethically reviewed and carried out in accordance with European Directive 2010/63/EU, in compliance with relevant guidelines (Italian Legislative Decree N. 26/2014) and the GSK’s policy and guidelines on the care, welfare and treatment of animals, in GSK animal facilities located in Siena, Italy (AAALAC accredited). The ethical protocol P004/26/01 was reviewed by the local GSK ethical committee. The study refers to the research project AWB 2020_05 approved by the Italian Ministry of Health.

Female CD1 mice, 7-week-old, were purchased from Charles River Laboratories and kept in a controlled environment (individually ventilated cages; 22 ± 3 °C; 12 h/12 h light/dark cycle). Our mice were commonly group-housed, so we only used females as they exhibit generally a reduced level of aggressiveness in medium to long housing conditions, which can affect scientific outcome^80^. To guarantee a more heterogenous and representative immune response, CD1 mice, which are outbred mice, were used in this study, instead of the more classical Balb/c or C57BL/6 inbred mice. Animals and their housing and husbandry were checked daily, and their well-being and health status were recorded in a dedicated logbook according to the local standard operating procedures. Final bleeding was performed under general anesthesia and animals were euthanized by cervical dislocation before recovery from anesthesia.

Mice were immunized intraperitoneally on Days 1, 29 and 57 with 10 µg/mouse of either of the three different batches of Ng GMMA (NgG1, NgG2 and NgG3) adsorbed to aluminum hydroxide (alum); 3 mg/ml), with 4CMenB (10 µg/mouse in terms of OMV component), or alum alone (10 animals per group; 200 µl/mouse). Sera were collected on Days 0, 56 (4wp2) and 72 (2wp3) and vaginal washes were collected on Day 74. In a different study using the same protocol, to evaluate the potential effects of LOS, animals were immunized with NgG or FA1090Δ*lpxl1*Δ*rmp*Δ*lgtF* GMMA (10 µg/mouse) adsorbed to alum (NgGΔ*lgtF*; 3 mg/ml), or alum alone (3 mg/ml).

### Luminex immunoassay for specific IgG and IgA detection

Luminex Magplex beads were activated according to the manufacturer’s instructions and incubated for 2 h with 40 µg/ml of Ng GMMA diluted in 500 µl of 50 mM MES. After washing twice with PBS-0.05% Tween 20, beads were resuspended in 500 µl of PBS-0.05% Tween 20 + 0.5% bovine serum albumin (BSA) (assay buffer) and stored at 4 °C. Test samples and a standard mouse pooled sera were prediluted in assay buffer and then consecutive threefold dilutions were performed (50 µl/well) in a 96-well microplate (Millipore Corporation). Standard and blank controls were included in each plate. An equal volume of conjugated microspheres (3000 beads/well) was added to prediluted samples and incubated for 60 min at RT in the dark on a plate shaker at 700 rpm. After washing with PBS, phycoerythrin (PE)-labeled secondary antibody was added to reveal specific IgA (50 µl/well of 5 µg/mL R-PE goat anti-mouse IgA, Southern Biotech) and IgG (50 µl/well of a 2.5 µg/ml R-PE goat anti-mouse IgG, Fcγ fragment specific, Jackson Immunoresearch) and the plate was incubated for 60 min with shaking. After washing, the beads were suspended in PBS and analyzed with Bioplex 200 system. Data were acquired with the Bio-Plex Manager software version 6.2 (Bio-Rad Laboratories). For each analyte, the median fluorescent intensity was converted to relative light units (RLU)/ml by interpolation from a five-parameter logistic standard curve. The final titer of each sample was expressed as geometric mean of concentrations with recovery 75–125% respectively to the median concentration of all the interpolated concentrations for each sample.

### Selection and characteristics of the strains used in functional assays

The rationale for the selection of relevant *N. gonorrhoeae* strains for the assay development and test was based on the analysis of the genetic variability of the outer membrane protein (OMP) components of Ng GMMA, with respect to a list of 59 of the more abundant OMPs through mass spectrometry analyses detection (listed in Supplementary Table 2). This list was used to define a multilocus typing schema (at protein sequence level) in a collection of more than 4000 sequenced genomes of gonococcal isolates and all strains were typed to assign protein allele identifiers and to measure a genetic distance. The resulting phylogenetic tree allowed the identification of 15 separate clusters (Supplementary Fig. 3). With the aim to present a hSBA panel representative of the general gonococcal population, single strains belonging to the most densely populated clusters (including clusters 3, 15, 4, 9 and 12) and multiple strains from the most genetically diverse cluster 1 were selected, also considering the existence of two PorB protein variants, 1a and 1b (Supplementary Table 4).

GC strains can be classified as serum-sensitive or serum-resistant depending on their susceptibility to killing by normal human serum (NHS). The strains tested in hSBA were evaluated for their complement sensitivity to non-immune human serum used as complement source during the development of the assay. Four out of eleven strains were classified as serum sensitive and therefore have been tested in hSBA after growth in medium containing CMP-NANA (which allows LOS sialylation and confers serum resistance to the bacterium. Supplementary Table 4 lists features of the different strains used in our functional assays.

A standard method for GC strains LOS immunotyping is not reported in literature. In order to determine the prevalent epitopes of LOS expressed by the selected GC strains, an immunochemical characterization of LOS was performed by western-blotting analysis on bacterial lysates grown in SBA-like condition. The following anti-LOS mAbs extensively described in literature were used: mAb 17-1-L1 (henceforth referred to as mAb L1), 4C4, L3,7,9 and 2C7. The tissue culture supernatant containing anti-LOS mAbs L1 was provided by Professor Sanjay Ram (Division of Infectious Diseases and Immunology, University of Massachusetts Medical School). Mouse mAb 2C7 was produced as recombinant internally. In particular the published variable region sequences were fused to the constant region of murine IgG2a^64^. Purified mAb 4C4 and supernatant of mAb L3,7,9 are commercially available. Results are summarized in Supplementary Table 5. The data showed that the strain panel was also heterogeneous in terms of LOS structures.

### Human serum bactericidal assay (hSBA)

Functional antibodies were measured by hSBA against the homologous FA1090 strain and the panel of heterologous strains described in the former section. Bacterial colonies from an overnight culture were resuspended in GC + 1% Isovitalex (CMP-NANA was added to the broth medium for serum-sensitive strains: 0.5 µg/ml for F62 and GC14 strains, 0.2 µg/ml for WHO-M and MS11 strains) and incubated at 37 °C with gentle shaking until the culture reached OD_600_ = 0.5. The broth culture was then diluted 1:10,000 in SBA buffer (Dulbecco’s phosphate-buffered saline [dPBS] + 1% BSA + 0.1% glucose) with the exception of the BG27 strain that was diluted 1:2500. Mouse sera, previously heat-inactivated at 56 °C for 30 min, were serially diluted in SBA buffer. The assay was assembled in a sterile 96-flat bottom well microplate in a final volume of 32 µl/well. The serial dilutions of each test sample were let to react with pre-diluted bacteria and with human serum from healthy donors, screened for lack of activity against GC strains, as complement source (16% for FA1090, BG27, BG8, SK92-679, WHO-F, WHO-G, WHO-N and 10% for F62, MS11, WHO-M, GC14). Human serum was obtained from clinical study MENB REC 2ND GEN-074 (V72_92) after subjects written consent, according to Good Clinical Practice and to the declaration of Helsinki.

The reaction mixture was incubated at 37 °C for 60 min at 160 rpm, then agar overlay medium was added to each well and the plate was incubated overnight at 37 °C with 5% CO_2_ in humid atmosphere. One day later, colony-forming units (CFUs) were automatically acquired with a high-throughput image analysis system (Discovery v12 Axiolab) and were automatically counted for each well by an image analysis system (Reading AxioVision). Bactericidal titer was defined as the reciprocal of the serum dilution giving a killing >50% respect to the average number of CFU calculated in replicates of ‘without serum’ control.

### Bacterial adhesion inhibition assay (BAI)

A cell-based fluorescent BAI assay was used to assess the capacity of alum-, 4CMenB-, NgGΔ*lgtF*- and NgG-immunized murine pooled sera (heat-inactivated) to inhibit the adhesion of homologous FA1090 and heterologous SK92-679 strains to Ect1 human ectocervical cells and SV-HUC1 human urethral epithelial cells.

Cultured cells were detached from 175 cm^2^-flask, after resuspension to avoid cell clumping. Cell number and viability were determined by an automated counter. They were then seeded into 96-well plates (3 × 10^4^ cells/well) and cultured in F-12K Nut Mix medium (21127-022, Thermo Fisher Scientific) for SV-HUC1 cell line or keratinocytes epidermal growth factor medium (10724-011, Thermo Fisher Scientific) + 20% FBS for Ect-1 cell line till confluence. GC strains were harvested from a fresh overnight plate culture into 10 ml GC + 1% Isovitalex medium. Bacteria were grown at 37 °C under shaking till OD_600_ = 0.5, then resuspended in dPBS and labeled with Oregon Green dye for 15 min at 37 °C. Afterwards, bacteria were washed to remove excess of dye, resuspended in dPBS-1% BSA, and combined, at final OD_600_ = 0.1, with an equal volume of serially cell medium-diluted sera for 15 min at RT. Bacteria-sera complexes were added to cell plates and incubated for 1 h at 37 °C to allow bacterium-cell adhesion. After three washes with dPBS, samples were fixed for 20 min with 4% formaldehyde at RT and, after one washing step with dPBS, finally one volume of distilled water was added to each well. Plates were analyzed by Opera Phenix instrument (Revvity).

### Characterization of T-cell responses

Mice were immunized intraperitoneally on Days 1 and 29 with either 4CMenB (10 µg/mouse in terms of OMV protein content), NgG (10 µg/mouse), or alum alone (10 animals per group). Five mice/group were sacrificed 12 days after the second immunization and their spleens were collected and processed individually. Splenocytes were isolated and seeded in round-bottomed 96-well plates at a density of 1.5 × 10^6^ cells/well. They were then stimulated overnight at 37 °C in RPMI containing 10% FBS, 1% penicillin, streptomycin, glutamine, 100 mM β-mercaptoethanol and anti-CD28 (BD, 2 µg/ml) with medium alone, heterologous *Salmonella* GMMA (kindly provided by the GSK Vaccines Institute for Global Health, Siena, Italy), Ng GMMA, SK92-679 GMMA, heat-killed (HK) FA1090 strain, HK SK92-679 strain or OMV from 4CMenB vaccine (OMV). Brefeldin A (5 µg/ml) was added for the last 4 h of incubation. Cells were then washed with PBS, stained with Live/Dead near infra-red (Invitrogen) for 20 min at RT, fixed and permeabilized with Cytofix/Cytoperm (BD) for 20 min at RT, washed with PBS and stored at 4 °C in PBS-1% BSA until analysis. For intracellular staining, the cells were washed with Perm/Wash Buffer 1X (BD) and incubated for 20 min in the presence of anti-CD16/CD32 Fc block (BD) and finally stained for 20 min at RT with anti-CD3-APC (Biolegend), anti-CD4-BV510 (Biolegend), anti-CD8-PE-CF594 (Beckton-Dickinson), anti-CD44-V21 (Beckton-Dickinson), anti-IFN-γ-BV785 (Biolegend), anti-TNF-α-PE (Miltenyi-Biotec), anti-IL-2-PECy5 (Biolegend), anti-IL-17-PECy7 (e-Bioscience), anti-IL-13-PerCPeFluor710 (Invitrogen), and anti-IL-4-PerCPeFluor710 (Invitrogen). After staining, the cells were washed with Perm/Wash buffer 1X, once with PBS, and finally resuspended in 150 µl of PBS. Samples were acquired on a LSRII flow cytometer (BD Biosciences) and data were analyzed using FlowJo software (Treestar). T-cell subsets were identified as follows: Th17 were all CD4^+^ T cells expressing IL-17; Th1 were all CD4^+^ T cells expressing IFN-γ, but not IL-17 and IL-4/IL-13; Th2 were all CD4^+^ T cells expressing IL-4/IL-13 but not IFN-γ and IL-17; Th0 were all CD4^+^ T cells expressing IL-2 and TNF-α and combinations of IL-4/IL-13 and IFN-γ.

### Statistical methods

The hSBA and Luminex data were evaluated using a one-way ANOVA model, considering group as a fixed factor and allowing for heterogeneous variances. Log2 and Log10 titers were analyzed respectively, with arithmetic means back-transformed into geometric mean titers (GMT) and differences in arithmetic mean, estimated from the same variance analysis model, into geometric mean ratio (GMR). Graphs were created showing individual values, geometric means, and 95% confidence intervals. For BAI assay data, it was checked if the response was at least 30% inhibition for each lot and test. Due to high variability, each lot was tested at least in two independent tests and additional tests were performed to strengthen the results when they appeared borderline. Data from all tests were analyzed by combining all results together and individually to evaluate consistency. Further, to combine all the tests, the mean of the response among the two replicates (within plate) at each dilution was computed for all the tests and data were analyzed all together. The dose-response curve linear part was identified, and the *p*-value of the regression was computed. The dose-response was considered significant if the *p*-value was equal or lower than 0.05. The overall mean of the responses at each dilution was compared to the 30% success criterion. For intracellular staining, immunization groups were compared with one-way ANOVA separately for each in vitro stimulation, and multiple comparison was performed with the Kruskal Wallis test.

## Supplementary information

Supplementary document

## Data Availability

The data that support the findings of this study are not openly available due to company restrictions but are available from the corresponding author upon reasonable request. Data are located in controlled access data storage at GSK.
