# Supplementary material for: A novel GMMA-based gonococcal vaccine demonstrates functional immune responses in mice
Source: NPJ Vaccines. 2025 Jul 5;10:146. doi: 10.1038/s41541-025-01190-1 (PMC12228689; doi:10.1038/s41541-025-01190-1)
Supplement: Supplementary file 1 — Supplementary document [file 41541_2025_1190_MOESM1_ESM.pdf]

Supplementary document

Supplementary Figure 1. Cellular-mediated immune response analysis. CD1 mice (5 animals/group) were immunized intra-peritoneally, twice, three weeks apart with Alum, NgG or 4CMenB. Fourteen days after the second immunization, the frequency of antigen-specific cytokine-secreting CD4+ T cells was determined by flow cytometry on splenocytes stimulated *in vitro* with *Salmonella* GMMA, FA1090 GMMA, FA1090 heat-killed (HK), SK92-679 GMMA, SK92-679 HK, or MenB OMV. Antigen-specific $CD4^+$ T cells are expressed as percentage of $CD4^+$ T cells. Th0 (A), Th1 (B), Th17 (C) and Th2 (D) cells subsets were identified based on the type of secreted cytokines as reported in the material and methods section. Total antigen-specific T cells (E) represent all the cytokine positive $CD4^+$ T cells. Single mice frequencies were graphed together with the median. Statistical analysis was performed comparing different immunization groups separately for each stimulation. Non-parametric Anova, Kruskal Wallis comparison, * $p_{value} < 0,05$

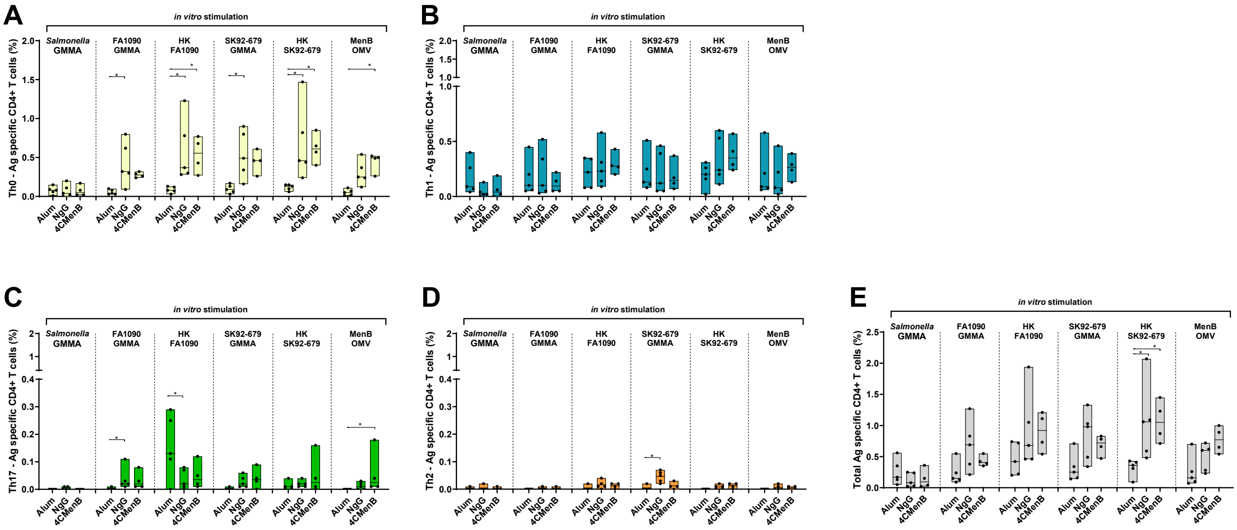

Supplementary Table 1. GMMA characterization: The table presents the analysis of different Ng GMMA batches, including NgGMMA1, NgGMMA2, NgGMMA3 derived from FA1090 Δ*lpxl1*Δ*rmp*, and NgGMMA-Δ*lgtF* from FA1090 Δ*lpxl1*Δ*rmp*Δ*lgtF*. Characterization includes the hydrodynamic radius measured by SEC-MALS (Size Exclusion Chromatography-Multi-Angle Light Scattering), purity percentage via FLR (Fluorescence Recovery after SE-HPLC), DNA content relative to protein concentration (µg DNA/µg protein), lipooligosaccharide (LOS) content (nmol OS/mg protein), and mAb 2C7 recognition status

	Radius	Purity	DNA content	LOS	
	**nm (SEC-MALS)**	**FLR (SE-HPLC)**	**µg DNA/µg protein**	**nmol OS/mg protein**	**mAb 2C7 recognition**
NgGMMA1	35.9	99%	0.003	244	yes
NgGMMA2	35.9	99%	0.002	268	yes
NgGMMA3	34.8	99%	<0.001	270	yes
NgGMMA-Δ*lgtF*	42.8	98%	0.003	134	no

Supplementary Figure 2. SDS-PAGE Analysis: Lane M, Molecular Weight Marker; Lanes 1-3, Ng GMMA samples from three different batches of Ng GMMA (NgGMMA1, NgGMMA2, NgGMMA3); Lane 4, GMMA derived from the strain FA1090 Δ*lpxl1*Δ*rmp*Δ*lgtF* (NgGMMA Δ*lgtF*)

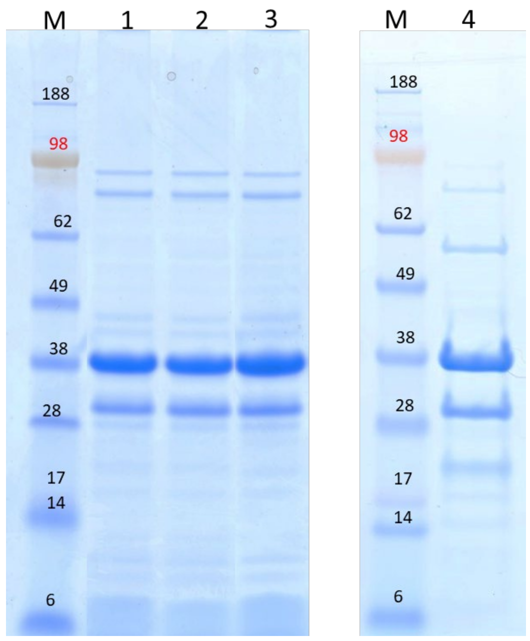

Supplementary Table 2: 59 Protein components identified as present in OMVs blebbed from FA1090 Δ*lpxl1*Δ*rmp* by mass spectrometry

UNIPROT code	NGO code	Protein name (full)	Protein name
Q5F5V7	NGO1812	Membrane protein	PorB 1B
Q5F6N6	NGO1513	Opacity protein opA54	Opa D
Q5FA01	NGO0233	Membrane protein	NspA
Q5F5P4	NGO1873	Peptidoglycan-binding protein LysM	NGO1873
Q5F5W8	NGO1801	Outer membrane protein assembly factor BamA	BamA
Q5F7V4	NGO1063	Membrane-bound lysozyme-inhibitor of c-type lysozyme family protein	MliC
Q5F501	NGO2139	Lipoprotein	Met Q
Q5FAD2	NGO0094	Type IV pilus biogenesis and competence protein PilQ (Outer membrane protein Omc)	NGO0094
Q5F848	NGO0948	Uncharacterized protein	NGO0948
Q5F5Y8	NGO1780	Outer membrane protein assembly factor BamE	BamE
Q5F9W0	NGO0277	Outer membrane protein assembly factor BamD	BamD
Q5F7X2	NGO1043	Lipoprotein	NA
Q5F9M1	NGO0372	Amino acid ABC transporter substrate-binding protein	NA
Q5F809	NGO0994	Lipid-modified azurin protein	Neisserial azurin
Q5F8C4	NGO0861	Uncharacterized protein	NGO0861
Q5F7F3	NGO1225	Peptidyl-prolyl cis-trans isomerase (EC 5.2.1.8)	MIP
Q5F8T2	NGO0678	Uncharacterized protein	
Q5F6A4	NGO1656	Peptidylprolyl isomerase (EC 5.2.1.8)	NGO1656
Q5FA17	NGO0217	Iron ABC transporter substrate-binding protein	NA
Q5F912	NGO0595	Pilus assembly protein PilW	NA
Q5F5F6	NGO1067	Adhesin MafA 1/4	MafA
Q5F652	NGO1714	Peptidylprolyl isomerase (EC 5.2.1.8)	NA
Q5F7W0	NGO1056	Peptidase	NA
Q5F7C7	NGO1251	Prokaryotic membrane lipolipid attachment site family protein	NA
Q5F6H4	NGO1393	Adhesin MafA 2/3	MafA 2/3

Q5F6Q5	NGO1494	Putrescine-binding periplasmic protein	
Q5F576	NGO2054	Spore cortex protein	NGO2054
Q5F7L8	NGO1152	ABC transporter substrate-binding protein	NGO1152
Q5F649	NGO1717	DSBA oxidoreductase	NGO1717
Q5F726	NGO1363	Multidrug transporter	MtrE
Q5FA63	NGO0168	ABC transporter substrate-binding protein	MntC
Q5F6J5	NGO1559	Membrane protein	OmpA
Q5F520	NGO2119	Transporter	NA
Q5F5E4	NGO1985	Hemolysin	936
Q5F845	NGO0952	TonB-dependent receptor	NA
Q5F651	NGO1715	LPS-assembly protein LptD	NGO1715
Q5F5W7	NGO1802	Membrane protein	NA
Q5F537	NGO2099	Prokaryotic lipo-attachment site family protein	NA
Q5F731	NGO1358	Glutamate dehydrogenase	NA
Q5F568	NGO2068	Uncharacterized protein	NA
Q5F6V7	NGO1438	Thiol:disulfide interchange protein	NA
Q5F8S0	NGO0690	Lipoprotein	NA
Q5F5B5	NGO2014	ABC transporter substrate-binding protein	NA
Q5F505	NGO2135	Lytic murein transglycosylase	NA
Q5F8Y3	NGO0626	Murein transglycosylase	NA
Q5F7H3	NGO1205	Ligand-gated channel	ZnuD/TdfJ
Q5FA28	NGO0206	Putrescine-binding periplasmic protein	
Q5F6Q7	NGO1492	Phospholipase A1	NA
Q5F5E8	NGO1981	Uncharacterized protein	ACP
Q5F8E4	NGO0834	Membrane protein	NGO0834
Q5F7G3	NGO1215	Uncharacterized protein	NA
Q5F6Q4	NGO1495	Ligand-gated channel	TbpA
Q5F5I3	NGO1942	Uncharacterized protein	NA
Q5F765	NGO1321	Uncharacterized protein	NA

Q5F9H8	NGO0416	Periplasmic protein	NA
Q5F7T9	NGO1081	CMP-N-acetylneuraminate-beta-galactosamide-alpha-2	GC-8
Q5F823	NGO0978	Thiol:disulfide interchange protein DsbD (EC 1.8.1.8) (Protein-disulfide reductase) (Disulfide reductase)	NA
Q5F932	NGO0572	Peptidase S41	NA
Q5FA29	NGO0205	Outer-membrane lipoprotein carrier protein	

Supplementary Table 3. Primers used in this study

Name	Sequence	Restriction site
Lpx UP Fwd	GGCATTTGTATTTTGCCGTCTG	
LpxL1 DO Rev	CGCCATTTTCTACGCTTTGCCAAG	
LpxL1 est FW	CCGCCAAACTCAATCCTTCG	
LpxL1 est REV	GCAAACTTTTGTTTCACCGTTTCCG	
UpIII-FOR	gctctagaGGTCGTCTATCCGTTCCGTA	XbaI
UpIII-REV	tcccccgggCTCAACGCCTGAAAACAACC	SmaI
DpIII-FOR	tcccccgggTCAAGCGCAAATGACTCAAG	SmaI
DpIII-REV	cccgctcgagGGGAAAGGCGTGAATTTGTA	XhoI
EryR_gono_SmaI-Fw	ATTCGCCCGGGAAACTTAAGAGTGTGTTGATAGTG	SmaI
EryR_gono_SmaI-Rev	ATTCGCCCGGGACCTCTTTAGCTTCTTGG	SmaI
UP_CHECK_NGO1577-Fw	GTGTGTCCAGTCGTAGCAGG	
DW_CHECK_NGO1577-Rev	AGGGATGATGATAAAACCATATCC	
LgtF-UP-fw	ATAGGGGAATTGTGCTCGAGACGCACCACAACGACAGTATGGAAAG	
lgtF-UP-rv	TCCTTCAGACGGCATTCCCGGGGGTTTCTCAAAGCATTTTGGTTTC	
lgtF-DO-fw	GGATCCCCATGGATACCCGGGCAAACTATATTATCTGTACAAATCC	
lgtF-DO-rv	AATTAAGTCGCGTTATCTAGAGTATCGATACCGTTATGAACAATCC	
cloKOF	ATGCCGTCTGAAGGATCCGTCAACCGTGATATAGATTGAAAAGTG	
cloKOR	TATCCATGGGGATCCGATCCACGCGTCTTAAGGCGG	

pETseqRv	GATATCCGGATATAGTTCCTC	
lgtF-ext-F	AAGACATCGGCCGGATTAATC	
lgtF-ext-R	GACGGTAAAAAAACGGCTGTCGG	

Supplementary Figure 3. Clustering analysis of GC strains

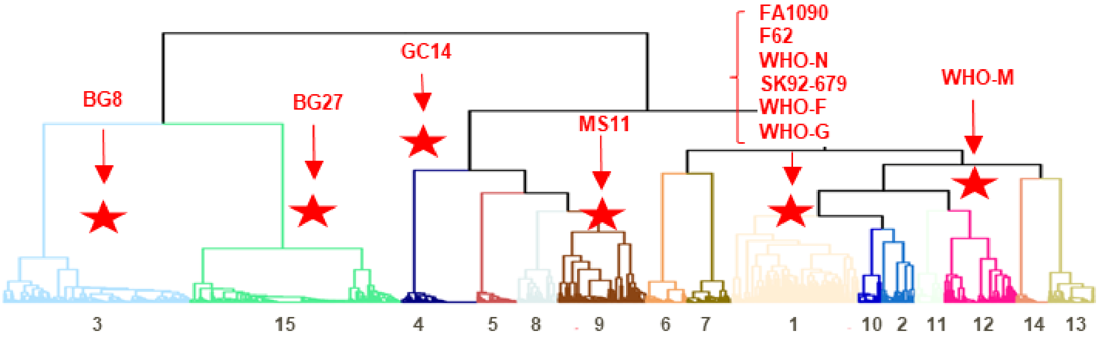

Fifteen clusters were identified by typing of 59 proteins from GC GMMA FA1090 Δ*lpxL1*Δ*rmp* and analyzing the genome of more than 4,000 gonococcal isolates. A red star indicates clusters for which a functional assay is available with the name of the strains. Each cluster is identified by a number reported at the bottom of the tree.

Supplementary Table 4. Features of GC strains used in functional assays

Strain	PorB variant	Cluster #	Serum resistance	Country	Source	Reference
FA1090	1b	1	YES	N/A	Boston University	
F62	1b	1	NO	N/A	ATCC (BAA-1837)	
MS11	1b	9	NO	USA	ATCC (BAA-1833)	
BG27	1b	15	YES	UK	Bristol University	
WHO-M	1b	12	NO	Australia	NCTC (13481)	Ref1
BG8	1b	3	YES	UK	Bristol University	
GC14	1b	4	NO	Canada	GSK	
SK92-679	1a	1	YES	USA	ATCC (BAA-1846)	
WHO-F	1a	1	YES	Canada	NCTC (13477)	Ref1
WHO-G	1a	1	YES	Thailand	NCTC (13478)	Ref1
WHO-N	1a	1	YES	Australia	NCTC (13482)	Ref1

Ref1: The novel 2016 WHO Neisseria gonorrhoeae reference strains for global quality assurance of laboratory investigations: phenotypic, genetic and reference genome characterization.
https://academic.oup.com/jac/article/71/11/3096/2462052

Supplementary Table 5. LOS immuno-characterization of GC strains used in functional assays

Strain	LOS immuno-characterization by Western-blot (mAbs recognition)			
	**2C7**	**L 3,7,9**	**4C4**	**L1**
WHO-F	YES	YES	NO	NO
WHO-G	NO	YES	YES	NO
WHO-M	NO	YES	NO	NO
WHO-N	YES	NO	NO	NO
F62	NO	YES	NO	NO
FA1090	YES	NO	NO	NO
GC14	NO	NO	NO	NO
MS11	YES	YES	NO	NO
SK92-679	YES	NO	NO	NO
BG27	YES	NO	NO	NO
BG8	NO	YES	NO	NO
